# Effects of a 6 Week Yoga Intervention on Executive Functioning in Women Screening Positive for Adult ADHD: A Pilot Study

**DOI:** 10.3389/fspor.2022.746409

**Published:** 2022-02-24

**Authors:** Kathryn Fritz, Patrick J. O'Connor

**Affiliations:** ^1^Department of Kinesiology, Temple University, Philadelphia, PA, United States; ^2^Department of Kinesiology, University of Georgia, Athens, GA, United States

**Keywords:** yoga, ADHD, cognitive performance, mood, mindfulness

## Abstract

**Purpose:**

Little is known about the effects of yoga training in adults with ADHD symptoms. This pilot study sought to determine the feasibility and selected psychological effects of 6 weeks of yoga training in women screening positive for adult ADHD compared to a wait-list control group.

**Methods:**

A randomized trial was conducted with 32 adult women (18–24 years) who volunteered after screening positive for adult ADHD as assessed by the Adult ADHD Self-Report Scale (ASRS). Participants were randomized to 6 weeks of Bikram yoga training or to a wait-list control group. The yoga intervention consisted of two 90-min classes per week. Multilevel models were used to test hypothesized interactions of yoga-induced improvements compared to controls across time (baseline, 3 weeks, and 6 weeks). The primary outcomes assessed inhibitory control, cognitive flexibility and working memory using the NIH Toolbox. Separate models with trait mindfulness, trait anxiety and expectations for change in either attention or working memory as covariates tested whether these variables mediated the changes in the three measures of executive function. Secondary outcomes included mood, perceived functional impairment and motivation for, and hyperactivity during, the cognitive tests.

**Results:**

No adverse events were observed. Attendance averaged 91.7% among the 69% of the sample that did not dropout. No significant Group X Time interactions were found for any of the psychological outcomes and the null executive function findings were unchanged when including the covariates.

**Conclusion:**

Six-weeks of yoga training twice per week is potentially feasible for women experiencing ADHD symptoms, but an exercise stimulus of this duration and magnitude yields no beneficial cognitive or mood outcomes.

## Introduction

Adult attention-deficit/hyperactivity disorder (ADHD) is characterized by inattention, hyperactivity and impulsivity (American Psychiatric Association, 2013). Results from a survey of ~20,000 US. adults without a previous ADHD diagnosis indicated 6.2% screened positive for adult ADHD using the World Health Organization's Adult Self-Report Screening questionnaire (Able et al., 2007). ADHD is more often diagnosed in men than women (Kessler et al., 2006). This may be in part because men exhibit signs of hyperactivity while women commonly present with inattention (American Psychiatric Association, 2013).

ADHD symptoms in adults are associated with impairment in work and social life (Able et al., 2007; Alderson et al., 2007; Cortese et al., 2015). Executive function is often impaired in adults with ADHD (Marije Boonstra et al., 2005). Executive function has been proposed to have three major components which include working memory, set-shifting, and inhibition (Miyake et al., 2000). Reduced executive function is thought to contribute to the expression of ADHD symptoms (Barkley, 1997). Adults with ADHD are at increased risk for being diagnosed with other psychological disorders (Kessler et al., 2006) and appear to suffer from sub-clinical elevations in anxiety to a greater extent than those who are disorder-free. When anxiety levels are reduced with therapeutic drugs, cognitive performance is improved in adults with ADHD (Bloch et al., 2017).

Treatments for ADHD include cognitive-behavioral therapies (Solanto et al., 2010; Knouse et al., 2017) and prescription drugs (Faraone and Glatt, 2010). Stimulant drug treatments have documented efficacy for improving ADHD symptoms (Faraone and Glatt, 2010; Solanto et al., 2010; Knouse et al., 2017); however, accessibility to treatments can be limited, and negative side effects can produce increased anxiety and difficulty sleeping (Spencer et al., 2005). Studies have reported sub-optimal response rates, with ~30–50% of individuals not experiencing remission of their ADHD symptoms (Biederman et al., 2006; Medori et al., 2008). Adherence to medication treatments has been reported to be as low as 64% (Adler and Nierenberg, 2010). Alternative tools for managing symptoms of ADHD are needed and adoption of regular exercise represents one plausible approach.

Exercise training has been inadequately investigated as an intervention for adults with ADHD symptoms. These studies usually have investigated the effects of exercise training on ADHD symptoms in children and adolescents. Authors of a review reported that exercise training of 5–12 weeks was associated with several benefits, including improvements in cognitive functioning. Nevertheless, the weight of the available evidence was inadequate to draw strong conclusions (Neudecker et al., 2015). The reviewers did note that yoga improved attention to a greater extent in girls than boys in one study (Haffner et al., 2006).

Yoga involves physical, mental and spiritual practices and is increasingly popular in the United States. Yoga participation has increased from ~6.3% in 2002 to ~11.2% in 2012 (Clarke et al., 2015). There are three key commonalities among different yoga styles: regulated breathing (pranayama), physical postures (asanas) and mindfulness. Mindfulness has been defined as a mental state achieved by focusing one's awareness on the present moment, while calmly acknowledging and accepting one's feelings, thoughts and bodily sensations (Brown and Ryan, 2003).

There is evidence that short-term (8-weeks) yoga results in increased lower body strength and flexibility and improvements in arterial stiffness and glucose tolerance (Hunter et al., 2013a,b; Tracy and Hart, 2013). Most previous studies have focused on physical outcomes however, one uncontrolled study showed improvements in mindfulness scores after 8 weeks of yoga (Hewett et al., 2011).

Yoga interventions have been infrequently studied among those with ADHD (Jensen and Kenny, 2004; Peck et al., 2005). Results from studies investigating the effects of yoga training in adults without ADHD have found both physical and mental health benefits (Cramer et al., 2013, 2014; Gothe, 2015), such as improvements in anxiety, cognition, and increased mindfulness (Hewett et al., 2011; Cramer et al., 2013; Gothe, 2015). Mindfulness meditation, in the absence of yoga, has been shown to activate the brain (Chambers et al., 2009; Hölzel et al., 2011; Lomas et al., 2015) and improve several psychological outcomes, especially stress and anxiety (Hofmann et al., 2010). One meta-analysis of 39 studies found that mindfulness therapy was moderately effective for improving symptoms of anxiety (*g* = 0.63) (Hofmann et al., 2010).

Proposed mechanisms through which mindful meditation might produce beneficial psychological effects include increased regulation of behavior, emotions and attention (Hölzel et al., 2011). Mindfulness is conceptually similar to good executive function, including the sustained attention needed for monitoring events, the ability to inhibit unwanted thoughts, and the use of cognitive flexibility to return to a focus on breathing and being in the moment if the mind wanders (Brown et al., 2007). Yoga combines mindfulness with physical activity in the form of poses. If an individual is holding a more challenging pose, he or she may become aware of, and attend to, lower limb muscles contracting to maintain balance and even feel some discomfort or pain. Unlike other types of physical activity, such as cycling or jogging, being mindful during yoga can explicitly encourage the individual to view this muscle contraction as it is—a sensation from the muscles in the leg, caused by neurons being excited by neurochemical and mechanical forces. During mindfulness the person is instructed to view this as neither bad nor good, and the individual is encouraged to inhibit the desire to appraise such stimuli as “good,” “bad,” or “neutral.”

Regular physical activity, in the absence of mindfulness, can produce brain-based adaptations hypothesized to maintain and improve executive functioning in children and older adults (Kramer and Erickson, 2007; Prakash et al., 2015). A meta-analysis of the effect of yoga on cognition found that in 15 randomized trials, the strongest average effects of regular yoga practice were found for improvements in attention and processing speed (g = 0.29) and executive function (g = 0.27) (Gothe, 2015). Because these effects were found among apparently healthy adults, it is plausible that larger effects could be realized among individuals with elevated ADHD symptoms.

It is unknown if adults with elevated ADHD symptoms will adhere to a yoga program. Regular attendance at a yoga class requires good planning but adults with elevated ADHD symptoms have planning deficits. Perceived lack of time is a major barrier to regular exercise in healthy adults (Grubbs and Carter, 2002; Lovell et al., 2010) yet time management skills are impaired in adults with elevated ADHD symptoms and some cognitive-behavioral ADHD therapies are aimed at improving time management skills (Solanto et al., 2010).

The purpose of this study was to determine (1) the feasibility of 6-weeks of yoga training, (2) the effects of 6-weeks of yoga training on executive function, and (3) whether changes in executive function after 6-weeks of yoga were partially explained by 6-week changes in trait mindfulness, trait anxiety or expectations for physical activity to induce improvements in attention or working memory during the cognitive testing sessions. Given the typical cost of yoga, it was considered useful to determine if significant improvements could be detected after a shorter-term intervention of only 6-weeks. Previous research has shown other forms of exercise can result in significant improvements in anxiety and cognition within 6-weeks (Herring et al., 2012; Heisz et al., 2017). It was hypothesized that the yoga intervention would be feasible based on: (H1.1) success in recruiting 30 participants within 6-months, (H1.2) the absence of major injuries or other adverse events, (H1.3) a low dropout rate (mean ≤ 10%), and (H1.4) a mean rate of attendance of ≥75%. It was also hypothesized that, compared to controls, (H2) improvements in executive function would be observed after 6-weeks of yoga and partially explained by covariation resulting from either: (H3.1) 6-week increases in trait mindfulness, (H3.2) 6-week reductions in trait anxiety, increased expectations for improved (H3.3) attention or (H3.4) working memory at 3- and 6-weeks.

## Methods

### Study Design

The current study was a pilot randomized controlled trial examining the feasibility and psychological effects of 6 weeks of yoga, compared to wait list controls, in women with symptoms of ADHD. An *a priori* statistical power analysis using G^*^Power indicated that 32 participants provided statistical power of 0.80 for the hypothesized treatment Xtime interactions. This analysis assumed an alpha error of 0.05, a correlation of *r* = 0.80 between repeated measures across time based on a prior test-retest reliability study of two of the NIH toolbox executive function tasks in a sample of 268 healthy adults (Zelazo et al., 2014), and an evidence-based small effect size (partial *eta*^2^ = 0.02, converted to an *f*-value [of 0.15], the metric required in G^*^power) (Gothe, 2015).

### Recruitment

Potential participants were recruited *via* flyers posted on local campus bulletin boards, email listservs, announcements in lecture classes and word-of mouth. A total of 32 women were eligible and volunteered to participate after undergoing screening and provided baseline data. A total of 27 women of the 32 original participants completed all study procedures.

### Screening

Screening involved potential participants completing questionnaires *via* Qualtrics. Individuals were excluded based on the following criteria: (a) male, (b) age younger than 18 or older than 39 years, (c) score below 14 on the 6-item Adult ADHD Self-Report Scale for DSM-5 (Ustun et al., 2017), (d) meeting the Physical Activity Guidelines (Services UDoHaH, 2008) assessed by the International Physical Activity Questionnaire (Craig et al., 2003), (e) recent participation in yoga (defined as >3 times per month during the past month), (f) contraindications to exercise participation as defined by an affirmative answers to questions on the Physical Activity Readiness Questionnaire (Thomas et al., 1992), (g) regular use (more than once per week during the past month) of prescription medication except for birth control, (h) regular use (more than once per week) of non-prescription stimulant (e.g., pseudoephedrine) including high amounts of caffeine (>600 mg per day), (i) low “past week” anxiety mood scores (1 SD < norms for the anxiety scale of the Profile of Mood States), (j) self-perceived risk for heat-related illness, (k) history of dizziness, faintness or nausea in response to high environmental heat, (l) history of heat-related illness, (m) history of a health care professional recommending avoidance of high environmental temperatures, (n) history of chronic or acute back or neck injury, and (o) history of diagnosed hypotension (BP <90/60 mmHg).

#### Adult ADHD Self-Report Scale for DSM-5

The Adult ADHD Self-Report Screening Scale for DMS-5 (ASRS-5) is a 6-item survey that was used to screen for potential ADHD. With over 2,500 citations, the research supporting the reliability and validity of the ASRS-5 screener is too large to detail here (Kessler et al., 2005). The ASRS-5 is a widely used tool that closely reproduces the overall clinical evaluations made by well-trained clinicians. For example, when compared to the weighted prevalence rate reported for semi-structured diagnostic interviews for adult ADHD in a general sample (6.5%), the ASRS-5 screened 11.2% of the general sample as having adult ADHD (Ustun et al., 2017). The sensitivity, specificity, and positive predictive value of the ASRS-5 were 91.4, 96.0, and 67.3%, respectively.

### Primary Outcomes

The primary outcome measures were three key aspects of executive function (Miyake et al., 2000): inhibitory control, cognitive flexibility and working memory (Miyake et al., 2000). Cognitive tests were administered using NIH Toolbox software with stimuli presented to all participants on the same iPad using standard, recommended instructions (Gershon et al., 2013). Each test took 3–5 min to complete.

#### Inhibitory Control (Flanker) Task

Inhibitory control was measured using the Flanker Inhibitory Control and Attention Test. This task consisted of four practice trials and 20 test trials. Test-retest reliability for the NIH Toolbox Flanker Task in adults was reported to be 0.80 (Weintraub et al., 2013).

#### Cognitive Flexibility

Cognitive flexibility was measured using the Dimensional Change Card Sort test. Participants matched stimuli based on color or shape. Four practice trials were followed by 30 test trials. The test trials consisted of a pre-switch block of 5 trials (e.g., shape) followed by a post-switch block of 5 trials (e.g., color) and a final block of 30 mixed-block trials (shape and color) presented in a random order. Test-retest reliability for the NIH Toolbox Dimensional Change Card Sort test in adults was reported to be 0.88 (Weintraub et al., 2013).

For both the Inhibitory control and Cognitive flexibility tasks, test trials were scored by combining accuracy and reaction time (RT) data into a 0–10 scaled score consisting of two vectors each ranging from 0 to 5. The accuracy vector score for adults with >80% accuracy (achieved by all participants in the present study at all trials) equaled 2.5 +0.125 ^*^ Number of Correct Responses. The RT vector scores were Base 10 log transformed median RTs for the correct incongruent trials or the least frequently cued dimension (shape for some individuals, color for others) after discarding RT outliers (RT <100 ms or >3 SD) from each participant's mean. These RT scores were rescaled by the NIH Toolbox Software to a range of 0 to 5 so that smaller reaction times yielded higher scale scores and larger reaction times yielded lower scale scores (Fritz and Connor, 2016).

#### Working Memory

Working memory was measured using the List Sorting Working Memory test (Tulsky et al., 2014). Participants were presented with a series of stimuli (i.e., illustrated pictures of animals or foods), displayed on the iPad monitor for 2 s while the name of the stimulus was simultaneously read *via* a computer presented voice. The participant was then asked to recall each stimulus in a series, mentally reorder them from smallest to largest, and recite the names of the stimuli in this order. Participants started by sequencing a two-item string and with each correct response the string is increased by a single item (up to a maximum of a seven-item string). If the participant was unable to sequence the string correctly, she was provided with a second trial of the same number of items; the task was discontinued when the participant provided incorrect responses on two consecutive trials with the same number of items. Participants began with the 1-list version of the task (sequencing among a single category). After finishing the 1-list section, the 2-list section was completed (sequencing of both “food” and “animals” within a string). The participant was required to sort stimuli by category (foods first, then animals) prior to sequencing the stimuli in size order. For example, if the words egg, horse, apple, grapefruit, elephant, raspberry, dog could be presented, the correct response was “dog, horse, elephant” followed by the words “raspberry, egg, apple, grapefruit.” Participants began the 2-list section with a two-item string, which was increased by a single item with each correct response. They were provided a second trial of the same number of items in the string following an incorrect response and the task was discontinued when the participant provided two consecutive incorrect responses or when the participant correctly sequences all seven items. List Sorting scores were based on a sum of the total correct across both lists. Scores range from 0 to 28. Test-retest reliability for the NIH Toolbox List Sorting Working Memory test in adults was reported to be 0.77 (Weintraub et al., 2013).

### Potential Mediating Variables

#### Mindful Attention Awareness Scale (MAAS)-Trait

The Mindful Attention Awareness Scale (MAAS) is a 15-item questionnaire that measures trait and state mindfulness. The trait scale lists statements about everyday experiences (e.g., I find myself doing things without paying attention) with responses ranging from “1” (almost always) to “6” (almost never). Scores to each statement are added and averaged, with higher scores indicating more mindfulness (Brown and Ryan, 2003). The state scale, used as a secondary outcome, is a 5-item questionnaire used to assess mindfulness with questions such as “I was doing something without paying attention” in response to being instructed to “indicate to what degree you were having each experience described below” (Brown and Ryan, 2003). Responses to the 5 items range from “0” (not at all) to “6” (very much). All items are reverse scored so that higher scores indicate higher mindfulness and then averaged to yield a criterion score that can range from 0 to 6. The state version documented mindfulness during each of the three cognitive testing trials and was administered immediately after the last cognitive test was completed. The MAAS has been shown to have good internal reliability, with one study reporting Cronbach's alpha of 0.89 (MacKillop and Anderson, 2007).

#### Trait Anxiety

The trait portion of the State-Trait Anxiety Inventory (STAI) was used to document changes in trait anxiety. The 20 item scale queries anxiety frequency with responses that range from “1” (almost never) to “4” (almost always). Scores for trait anxiety range from 20 to 80 (Spielberger, 1983). Internal consistency and test-retest reliability for the trait portion of the STAI have been reported to be 0.89 and 0.88, respectively (Barnes et al., 2002).

#### Expectations

Expectations were assessed using a questionnaire designed to assess expected psychological outcomes of exercise. The nine-item item survey asks about expected benefits regarding: reduced anxiety, reduced depression, increased feelings of energy, decreased feelings of fatigue, reduced anger, reduced confusion, increased memory, decreased reaction time and increased sustained attention. Each outcome was preceded by the statement “A major benefit of physical activity for me is…”. Participants were asked to rate their agreement with each statement ranging from 1 “strongly disagree” to 5 “strongly agree” (Lindheimer et al., 2017). Expectation for improved memory was included as a covariate in the analysis that tested the working memory primary outcome. Expectation for improved attention was included as a covariate in the analyses that tested the inhibitory control and cognitive flexibility outcomes.

### Secondary Outcomes

#### Hyperactivity During the Cognitive Tests

Hyperactivity was assessed during the cognitive tests using Actigraph GTX3 accelerometers placed on the participant's non-dominate ankle and wrist. A sample rate of 100 Hz at 1 second epoch lengths was used to assess hyperactivity. Criterion activity count data was the average counts obtained starting from 30 s before the start of the cognitive tests to 30 s after the completion of the cognitive tests. Total activity counts were divided by time and expressed as mean counts/minute.

#### Motivation to Perform the Cognitive Tests

Motivation to perform the cognitive tests was documented immediately before each cognitive test using a 100 mm visual analog scale with the verbal anchors “no motivation” and “highest motivation possible.” Participants indicated their level of motivation to complete mental work using a sliding ruler on a line on the computer to indicate the level of motivation. This measure has been used in previous studies to assess motivation for cognitive work (Fritz and Connor, 2016; Boolani et al., 2017).

#### Perceived Functional Impairment

Perceived functional impairment was measured using the Barkley Functional Impairment Scale (BFIS) Quick Screen (6 item) (Barkley, 2011). Respondents were instructed to describe their difficulty in functioning during the past month at baseline, at week 3, and at week 6. The six items assess the severity (0–9) of perceived impairment averaged across six domains (family, friends, education, self-care, household management and work). Chronbach's alpha for the BFIS Quick screen has been reported to be 0.916, with test-retest reliability ranging from 0.40 to 0.72 (Barkley, 2016).

#### Mood

Mood was assessed using the Profile of Mood States-Brief Form. The POMS-BF is a 30 item questionnaire that assesses the intensity of six different moods: anxiety, depression, anger, confusion, vigor, and fatigue. Responses range from 0 (indicating not at all) to 4 (indicating extremely) (McNair et al., 2003). Participants were instructed to report how they had been feeling during the past 7 days. Internal consistency estimates for the POMS individual subscales have been reported to be: 0.91 for anxiety, 0.95 for depression, 0.92 for anger, 0.76 for confusion, 0.90 for vigor, and 0.94 for fatigue (Curran et al., 1995).

#### Isometric Leg Strength Test

Isometric knee extension and flexion strength was measured using a Biodex dynamometer. The participant sat upright on the seat of the machine, the axis of the dynamometer was adjusted to match the knee joint axis and the chest and leg were secured with straps. The researcher measured the distance from the top of the weight stack on the machine when the weight was at rest to the top of the weight stack when the participant was at full extension to ensure accurate strength calculations by having the participant complete one unweighted repetition. Participants then completed submaximal isometric contractions of her dominant leg at a knee joint angle of 60° (with full knee extension being 0°), followed by 45 s of rest. Participants held each contraction for a total of 3 s. This process was repeated twice for a total of six contractions. Participants then complete the same process again except that three maximal isometric repetitions were performed and the average served as the criterion score.

### Procedures

After providing written informed consent, participants completed all baseline measures. After 7 days, participants returned to the Exercise Psychology Lab and group assignment was made. A total of 32 participants were randomized to either a 6 week yoga training program or a wait list control by one of the authors (PJO) using a tool available at Randomizer.org. Participants allocated to the control group were asked to maintain their current physical activity levels and not to adopt a yoga training program until their participation in the study was completed. In an effort to increase adherence to the study protocol, participants in the control group were offered a free 6 week yoga training program after providing data for the 6 week study and complying with all study procedures. Participants in the intervention group began the yoga intervention after providing baseline data. Participants attended the yoga sessions at a local yoga studio. Studio check-in information provided attendance data. At the end of the 3rd and 6th week, participants in both groups visited the laboratory and completed all the baseline measures. Participants were emailed weekly reminders about complying with the study procedures and reminders about upcoming lab visits.

The yoga training program consisted of 180 min of yoga per week for 6 weeks, led by certified yoga instructors. Training sessions were separated by at least 48 h. Each session involved 26 yoga poses in the same room heated to ~105 degrees. Before and after each yoga session, participants completed questions which they accessed on their phones *via* Qualtrics using a link that had been emailed to them. Thirty minutes before and within 30 min after their yoga session, participants were asked to complete the state mindfulness scale and the POMS-BF using “right now” instructions (McNair et al., 2003). Within 30 min post-exercise, ratings of average overall perceived exertion and pain intensity experienced during the yoga session using Borg's 6–20 (Borg, 1982) scale and a 0–10 numerical-graphical pain intensity scale, respectively, were completed (Farrar et al., 2001).

### Statistical Analysis

Cognitive performance, questionnaire, accelerometry, and strength data were entered into IBM SPSS Statistics Version 23 and the accuracy double checked. During the last four yoga sessions there was a high amount of missing perceived exertion, pain intensity and mood data post-exercise (~50%). Less than 1% of the data were missing during the first eight yoga sessions and each participant attended at least one of the two planned sessions each week during the first 4 weeks. Thus, weekly averages are provided for the data generated in association with the yoga sessions across the first 4 weeks.

With regard to the 6-week data, an intent-to-treat approach was used and missing data (<1% total) were replaced by carrying forward the last observation. Descriptive data are presented as means and standard deviations. Multilevel models were fit in the R statistical computing software using the lme4 and lmerTest packages and *p* < 0.05 as the criterion for statistical significance to test the hypotheses. For each outcome, two models were fit. The first predicted the outcome with Group, Time, and the Group x Time interaction and allowed varying intercepts by participant. The second models included the same predictors for the primary outcomes and varying intercepts but added trait mindfulness, trait anxiety, and either expectation for improved attention or working memory as time-varying covariates.

## Results

### Participants and Descriptive Statistics

A total of 32 individuals met inclusion criteria and completed all baseline measures; 16 were assigned to the control condition and 16 to the yoga condition. No participants in the control group dropped out of the study. Participant characteristics are presented in Table 1.

**Table 1:** Participant characteristics (*N* = 32).

		**Mean**	**SD**	**Range**
Sample characteristic:	Age (years)	20.16	1.46	18–24
	BMI	22.82	2.64	18.46–29.35
	IPAQ total (MET-min/week)	2995.19	2407.75	39.60–8478.00
	IPAQ MVPA(MET-min/week)	1338.75	1582.79	0–6480.00
	POMS-tension	10.56	2.31	7.0–15.00
	Adult ADHD self-report scale for DSM-5 (ASRS-DSM5)	17.19	1.53	15.00–21.00

### Feasibility

Data were collected from October 12, 2017 to March 20, 2018. No injuries or adverse events were reported. Thirty-one percent of the yoga participants dropped out of the study. One participant dropped out for unknown reasons and others dropped out due to: illness (*n* = 1), perceived lack of time (*n* = 2) and graduation (*n* = 1). For the entire yoga group, session attendance averaged 77.6% (9 ± 3.2, range 4–12). For the eleven participants who did not drop out, session attendance averaged 91.7% (11 ± 1.3, range 8–12 sessions).

On average the yoga sessions were perceived as hard and moderately painful. Post-session, consistent small increases in energy and small reductions in tension were reported. During all but the first week, fatigue symptoms increased after yoga. Average perceptual and mood responses before and after yoga sessions during the first 4 weeks are presented in Table 2.

**Table 2:** Means (SD) and ranges for acute affective responses to the Bikram yoga sessions.

	**Week 1**		**Week 2**		**Week 3**		**Week 4**	
**Outcome variable**	**Pre**	**Post**	**Pre**	**Post**	**Pre**	**Post**	**Pre**	**Post**
**Tension**								
*M*	4.88	1.82	3.06	1.68	2.62	1.23	2.27	1.96
*SD*	2.57	1.33	2.80	2.03	2.27	1.00	2.49	1.72
Range	0–9.00	0–4.5	0–10.5	0–5.50	0–8.00	0–3.00	0–8.50	0–5.00
**Depression**								
*M*	3.44	0.73	1.53	0.59	2.35	0.86	1.04	0.91
*SD*	2.62	0.72	2.46	1.50	3.15	1.38	1.93	2.43
Range	0–9.00	0–2.00	0–9.50	0–5.00	0–9.00	0–4.00	0–6.00	0–8.00
**Anger**								
*M*	2.53	0.46	2.09	1.27	2.35	0.68	1.65	0.68
*SD*	2.83	0.72	3.03	2.32	2.23	1.25	3.14	1.31
Range	0–9.0	0–2.00	0–11.00	0–7.00	0–7.50	0–4.00	0–9.00	0–4.00
**Vigor**								
*M*	4.38	6.00	4.53	5.46	4.23	4.59	4.96	5.36
*SD*	2.95	2.85	3.03	2.38	3.35	1.70	2.59	2.67
Range	0–11.00	1.00–10.00	0–12.00	2.00–9.00	0–11.00	2.50–8.00	0–8.50	1.00–9.50
**Fatigue**								
*M*	6.16	5.41	4.16	5.46	3.85	5.64	3.08	4.59
*SD*	3.27	2.60	2.24	4.92	3.11	3.29	2.89	2.49
Range	1.00–11.50	0.50–9.00	0–9.50	0–13.00	0.50–10.00	0.50–13.00	0–10.00	1.00–8.00
**Confusion**								
*M*	4.57	3.27	3.46	3.05	4.12	3.82	4.04	3.18
*SD*	2.44	1.03	1.41	1.33	1.69	1.66	1.98	1.12
Range	1.00–9.00	2.00–5.50	1.00–10.5	2.00–6.00	2.00–7.00	2.00–7.50	1.00–8.00	1.50–5.00
**MAAS–state**								
*M*		4.14		4.37		4.67		4.51
*SD*		0.96		1.15		0.92		0.80
Range		2.10–5.40		2.10–5.80		3.00–5.80		3.00–5.80
**RPE (6–20)**								
*M*		15.46		13.96		14.73		14.00
*SD*		1.35		2.32		2.36		2.64
Range		13.50–17.50		11.00–18.00		11.00–19.00		10.50–19.00
**Pain**								
*M*		4.32		4.27		4.18		3.64
*SD*		2.47		2.26		2.65		2.30
Range		1.50–8.00		1.00–8.50		0.50–9.00		0–7.5

### Primary Cognitive Outcomes

Descriptive cognitive data are provided in Table 3. There were no significant group-by-time interactions for any of outcome variables for the Flanker Inhibitory Control and Attention test (*p* > 0.153), the Dimensional Change Card Sort test (*p* > 0.216), or the List Sorting Working Memory test (*p* > 0.305). These results were unchanged when including the covariates.

**Table 3:** Means (SD) for the NIH Toolbox Flanker, Dimensional Change Card Sort (DCCS) and list sorting working memory tests.

	**Yoga group**			**Control group**			**Effect size**
**Variable**	**Baseline**	**3-weeks**	**6-weeks**	**Baseline**	**3-weeks**	**6-weeks**	**6-week gain scores**
Flanker accuracy, 0–20	20.00 (0.00)	19.94 (0.25)	19.88 (0.50)	19.88 (0.34)	20.00 (0.00)	19.94 (0.25)	−0.55
Flanker raw reaction time (msec)	590.00 (170.00)	560.00 (120.00)	540.00 (110.00)	620.00 (190.00)	600.00 (200.00)	590.00 (210.00)	0.11
Flanker reaction time CV	0.16 (0.07)	0.17 (0.09)	0.20 (0.11)	0.21 (0.09)	0.21 (0.10)	0.24 (0.15)	0.18
Flanker age-adjusted/U.S. norms	94.31 (18.86)	96.13 (15.97)	98.81 (16.12)	91.13 (19.55)	95.81 (19.71)	98.38 (19.75)	−0.15
Flanker scaled score, 0–10	8.89 (0.78)	8.97 (0.58)	9.06 (0.56)	8.68 (0.93)	8.91 (0.83)	8.94 (0.92)	−0.11
DCCS accuracy, 0–30	29.63 (0.50)	29.81 (0.40)	29.69 (0.60)	29.44 (0.89)	29.19 (0.83)	29.63 (0.50)	−0.20
DCCS raw reaction time (msec)	570.00 (150.00)	540.00 (150.00)	530.00 (140.00)	610.00 (190.00)	600.00 (250.00)	590.00 (190.00)	0.06
DCCS reaction time CV	0.28 (0.08)	0.24 (0.08)	0.25 (0.08)	0.28 (0.08)	0.37 (0.33)	0.34 (0.16)	0.85
DCCS age-adjusted/U.S. norms	105.00 (16.96)	107.38 (16.89)	103.75 (15.37)	99.31 (17.16)	105.13 (15.62)	102.50 (14.37)	−0.28
DCCS scaled score, 0–10	9.05 (0.72)	9.17 (0.76)	9.05 (0.69)	8.76 (0.84)	9.02 (0.72)	8.97 (0.64)	−0.29
Working memory raw score	18.81 (2.26)	20.31 (2.39)	20.00 (2.56)	19.88 (2.68)	20.75 (2.62)	21.13 (2.16)	0.02
Working memory age-adjusted U.S. norms	100.69 (12.03)	108.06 (11.83)	106.38 (12.55)	106.13 (13.51)	110.06 (12.54)	112.19 (10.03)	−0.03

*CV, coefficient of variation*.

The mean, standard deviation, and 95% confidence interval for the yoga group for the Flanker computed score at Time 1 was 8.89 ± 0.78: 95% CI [8.47, 9.30], at Time 2 was 8.97 ± 0.58: 95% CI [8.66, 9.27], and at Time 3 was 9.06 ± 0.56: 95% CI [8.76, 9.36]. Equivalent data for the for the control group for the Flanker computed score at Time 1 was 8.68 ± 0.93: 95% CI [8.18, 9.17], at Time 2 was 8.91 ± 0.83: 95% CI [8.47, 9.35], and at Time 3 was 8.94 ± 0.92: 95% CI [8.45, 9.44]. The mean, standard deviation, and 95% confidence interval for the yoga group for the Dimensional Change Card Sort test computed score at Time 1 was 9.05 ± 0.72: 95% CI [8.67, 9.43], at Time 2 was 9.17 ± 0.76: 95% CI [8.76, 9.57], and at Time 3 was 9.05 ± 0.69: 95% CI [8.68, 9.42]. Equivalent data for the control group for the Dimensional Change Card Sort test computed score at Time 1 was 8.76 ± 0.84: 95% CI [8.31, 9.20], at Time 2 was 9.02 ± 0.72: 95% CI [8.64, 9.40], and at Time 3 was 8.97 ± 0.64: 95% CI [8.64, 9.31].

The mean, standard deviation, and 95% confidence interval for the yoga group for the List Sorting Working Memory test at Time 1 was 18.81 ± 2.26: 95% CI [17.61, 20.02], at Time 2 was 20.31 ± 2.39: 95% CI [19.04, 21.58], and at Time 3 was 20.00 ± 2.56: 95% CI [18.64, 21.36]. Equivalent data for the control group for the List Sorting Working Memory test at Time 1 was 19.88 ± 2.68: 95% CI [18.45, 21.30], at Time 2 was 20.75 ± 2.62: 95% CI [19.35, 22.15], and at Time 3 was 21.13 ± 2.16: 95% CI [19.98, 22.27].

### Secondary Outcomes

Descriptive mood, motivation, mindfulness, strength and hyperactivity data are presented in Table 4. There was a high percentage of missing leg hyperactivity data due to equipment malfunction.

**Table 4:** Means (SD) for questionnaire data, wrist hyperactivity during cognitive tasks, and leg strength data.

	**Yoga group (** ***n*** **=16)**			**Control group (*****n*** **=** **16)**			**Effect size**
**Variable**	**Baseline**	**3-weeks**	**6-weeks**	**Baseline**	**3-weeks**	**6 weeks**	**6-week gain scores**
Trait anxiety	47.31 (11.49)	46.56 (12.29)	45.00 (12.33)	44.00 (9.68)	42.56 (9.11)	42.69 (8.69)	0.09
POMS tension	3.69 (2.96)	3.69 (3.05)	2.69 (3.16)	3.19 (2.88)	2.88 (2.73)	3.56 (2.80)	0.46
POMS depression	1.63 (2.16)	1.88 (2.42)	1.38 (2.47)	2.06 (2.86)	1.75 (2.18)	1.94 (3.36)	0.05
POMS anger	1.13 (2.50)	2.38 (5.03)	0.94 (2.41)	0.69 (1.08)	0.94 (1.06)	0.69 (1.14)	0.10
POMS vigor	6.81 (3.66)	4.75 (3.99)	6.13 (4.24)	6.88 (2.22)	6.31 (3.03)	6.56 (3.44)	−0.12
POMS fatigue	4.94 (4.22)	4.88 (3.69)	4.06 (3.86)	4.94 (3.61)	7.06 (3.59)	7.25 (3.80)	0.82
POMS confusion	4.50 (3.12)	4.63 (3.32)	3.94 (3.42)	4.44 (1.90)	4.88 (2.34)	4.44 (2.13)	0.20
Mindfulness (trait)	3.25 (0.59)	3.25 (0.57)	3.53 (0.56)	3.22 (0.68)	3.17 (0.76)	3.16 (0.87)	0.50
Expectations	32.94 (5.95)	32.75 (3.80)	33.69 (4.16)	32.56 (4.79)	32.06 (5.14)	31.25 (7.25)	0.36
Functional impairment	2.90 (1.71)	2.63 (1.62)	2.43 (1.77)	3.79 (1.38)	3.71 (1.89)	3.26 (1.75)	−0.04
Motivation	72.88 (19.72)	72.19 (19.77)	74.25 (20.71)	59.38 (20.69)	54.19 (23.43)	53.94 (24.61)	0.32
Mindfulness (state)	4.22 (1.54)	3.81 (1.42)	4.07 (1.28)	3.43 (1.41)	3.24 (1.80)	3.30 (1.33)	0.01
Wrist hyperactivity (counts/min)	633.92 (518.86)	508.91 (447.80)	620.53 (326.97)	256.04 (180.99)	460.36 (298.86)	571.41 (273.20)	0.95
Quadriceps strength (N-M)	82.75 (6.35)		84.34 (5.85)	84.43 (4.23)		86.43 (6.07)	−0.07
Hamstring strength (N-M)	59.44 (3.25)		61.59 (2.86)	54.36 (2.91)		55.56 (2.69)	0.32

The condition-by-time interactions for all secondary outcome measures were not statistically significant (*p* > 0.05).

## Discussion

The present investigation examined the feasibility and effects of yoga on executive function in women with symptoms of ADHD. No participants in this study reported any major injuries or adverse events due to participating in the yoga; this finding is consistent with the general consensus that participation in yoga is generally considered safe (Barnes et al., 2002). The attendance at the yoga classes for those who did not dropout (11/12, 91.7%) was generally consistent with class attendance rates reported in prior yoga studies. For example, median class attendance in trials requesting attendance at 12 yoga sessions was eight (Saper et al., 2004) in samples with back pain. The biggest feasibility concern was the high dropout rate of ~31%. Results from a recent meta-analysis of 168 randomized control trials estimated the average dropout rate to be 11.4% for yoga interventions (Cramer et al., 2016). Thus, women with symptoms of ADHD appear to be at higher risk of dropout from yoga programs. This is consistent with the high rate of non-adherence to medication treatment among adults with ADHD. Future research is needed to learn if strategies not used in the present study can minimize dropout from yoga in this population.

Results from the current study showed no effect of 6-weeks of yoga on any of the measures of executive function. Small sample bias cannot be ruled out as contributing to the null findings. It is also possible that 6-weeks of yoga truly has no sustained effect on executive function in adult women with ADHD. Results from a meta-analysis (Gothe, 2015) indicated yoga training has a small (*g* = 0.27) but significant effect on improving executive function performance. However, participants in the studies analyzed were older, on average, than the participants in the current sample (~45 years old vs. 20 years old). Cognitive abilities decline with age (Bherer et al., 2013) and older adults may be better able to experience improvements in executive function performance after completing yoga training in part because their baseline might be lower. The potential moderating effect of age was not considered in the meta-analysis (Gothe, 2015).

Another possible explanation for the null findings in the current study is the properties of the cognitive tests used (i.e., ceiling effects and sub-optimal sensitivity to change) combined with the relatively high level of cognitive performance in our sample. Participants in both groups at the baseline had nearly perfect accuracy scores on the Flanker Inhibitory Control and Attention test and the Dimensional Change Card Sort test. Perhaps due to the extensive exclusion criteria, the sample tested may have consisted largely of high functioning individuals with ADHD symptoms, a sub-group that is garnering increased attention (Lesch, 2018). The scoring rubric for List Sorting Working Memory test used may have been inadequately sensitive to change and contributed to the null findings. Participants are either “correct” or “incorrect” for a given string of items; however, a significant difference may have been detected if the number of correct items remembered in a given string were used in the scoring protocol.

It is plausible that each yoga bout had a transient effect on improving executive function similar to the effects of acute exercise. The cognitive testing was performed on days in which the participants had not engaged in yoga. Consequently, the cognitive testing may have been timed in a way that missed true acute effects of yoga.

Our assessment of lower body strength revealed little difference in performance between the two groups. Results from one previous study investigating the effects of 8 weeks of yoga on fitness outcomes in young adults revealed significant changes in lower leg strength in the yoga group but not the control group (Tracy and Hart, 2013). We cannot rule out that the yoga intervention used in the current study was insufficient in volume to produce significant changes in lower body strength.

It was unexpected that trait anxiety and mood states were not significantly changed in the yoga group given the benefits for anxiety and depression reported in other yoga studies (Woolery et al., 2004; Cramer et al., 2013). A previous study investigating the effects of twice weekly Iyengar yoga reported significant decreases in both trait anxiety and depression for yoga participants after five weeks when compared to wait-list control group (Woolery et al., 2004). We speculate that some unmeasured characteristic of participants contributed to this sample's resistance to anxiolytics with yoga training such as a chronic psychiatric (depression, parental ADHD, substance abuse) or medical (anemia, asthma, sleep apnea) comorbidity (Chen et al., 2019).

The intervention used in the current study did not result in significant changes in trait or state mindfulness scores in the yoga group. However, by the end of the 6 weeks trait mindfulness scores for the yoga group had improved slightly and the control group had decreased slightly, resulting in a non-significant, moderate effect size (0.50). Mindfulness based interventions, in both healthy individuals and those with ADHD, that have found significant positive changes in mindfulness and other psychological outcomes have included an at-home practice as part of the intervention (Maryanna et al., 2008; John et al., 2013; Bueno et al., 2015; Canby et al., 2015). This could potentially explain why mindfulness scores were not improved during the 6 weeks of yoga. It could be that the yoga participants in the current study did not have enough practice using concepts of mindfulness to produce a significant change. Two previously conducted 8-week mindfulness-based intervention studies, that did include at-home practice, have found significant decreases in self-reported adult ADHD symptoms of inattention and hyperactivity/impulsivity (John et al., 2013; Bueno et al., 2015). One of these studies found significant improvements in cognitive performance in adults with ADHD and healthy controls (Bueno et al., 2015) but the other did not (John et al., 2013). Mindfulness was not assessed in either of these studies; thus, it is not possible to know if changes in mindfulness across the 8 week interventions utilized can explain the changes in cognitive performance.

The current pilot study had several limitations. The sample size was small and there was a larger than average dropout rate. Women were selected for the present study since women have largely been excluded from studies investigating the effect of exercise on symptoms of ADHD and report higher levels of yoga participation when compared to men (Cramer et al., 2016). Thus, one potential limitation is the generalizability of the results to adults diagnosed with ADHD and men experiencing ADHD symptoms. Studies comparing the consequences of yoga in samples of women diagnosed with ADHD to those with elevated ADHD symptoms in the absence of a diagnosis have not yet been conducted.

In conclusion, yoga training is potentially feasible for women experiencing ADHD symptoms. There were no observed significant changes in cognitive performance, mood, mindfulness or motivation in the yoga group when compared to the control group. It is plausible that a yoga intervention of longer duration with more frequent yoga session per week would prove more beneficial for this population. Future studies should aim to determine what strategies can improve adherence to yoga programs and if positive psychological effects are experienced in women with elevated ADHD symptoms if they perform yoga at a higher weekly frequency or for a longer duration.

## Data Availability Statement

The original contributions presented in the study are included in the article, further inquiries can be directed to the corresponding author.

## Ethics Statement

The studies involving human participants were reviewed and approved by Human Research Protection Program; University of Georgia. The patients/participants provided their written informed consent to participate in this study.

## Author Contributions

Both authors listed have made a substantial, direct, and intellectual contribution to the work and approved it for publication.

## Funding

This study received funding from the Department of Kinesiology's Kindig Scholarship at the University of Georgia and Pure Action Inc. Neither funder was involved in the study design, collection, analysis, interpretation of data, the writing of this article, or the decision to submit it for publication.

## Conflict of Interest

This study received funding from the Pure Action Inc. The authors declare that the research was conducted in the absence of any commercial or financial relationships that could be construed as a potential conflict of interest.

## Publisher's Note

All claims expressed in this article are solely those of the authors and do not necessarily represent those of their affiliated organizations, or those of the publisher, the editors and the reviewers. Any product that may be evaluated in this article, or claim that may be made by its manufacturer, is not guaranteed or endorsed by the publisher.
